# Treatment effects of fibrinogen concentrates vs. cryoprecipitate for correcting hypofibrinogenemia in cardiac surgery patients: a systematic review and meta-analysis

**DOI:** 10.3389/fcvm.2025.1671405

**Published:** 2025-10-17

**Authors:** Yue Chen, Wenjie Yang, Chongyang Zhao, Fujue Wang, PengWei Ren, Xuemei Liu, Tingyong Cao, Shuming Ji, Lei Chen, Deying Kang

**Affiliations:** ^1^ Clinical Trial Center, West China Hospital, Sichuan University, Chengdu, Sichuan, China; ^2^ Center of Biostatistics, Design, Measurement and Evaluation (CBDME), West China Hospital, Sichuan University, Chengdu, Sichuan, China; ^3^ Department of Evidence-based Medicine and Clinical Epidemiology, West China Hospital, Sichuan University, Chengdu, China; ^4^ State Key Laboratory of Biotherapy, West China Hospital, Sichuan University, Chengdu, China; ^5^ Department of Pulmonary and Critical Care Medicine State Key Laboratory of Respiratory Health and Multimorbidity, Targeted Tracer Research and Development Laboratory, Med-X Center for Manufacturing, Frontiers Science Center for Disease-related Molecular Network, West China Hospital, West China School of Medicine, Sichuan University, Chengdu, China; ^6^ Editorial Department of Chinese Journal of Clinical Thoracic and Cardiovascular Surgery, West China Medical Publisher, West China Hospital, Sichuan University, Chengdu, China; ^7^ Department of Hematology, The Second Hospital & Clinical Medical School, Lanzhou University, Lanzhou, Gansu, China; ^8^ Department of Neurology, West China Hospital, Joint Research Institution of Altitude Health, Sichuan University, Chengdu, China

**Keywords:** hypofibrinogenemia, cardiac surgery, cardiopulmonary bypass, fibrinogen concentrates, cryoprecipitate, meta-analysis

## Abstract

**Background:**

Hypofibrinogenemia in cardiac surgery increases bleeding risk, but the efficacy and safety of fibrinogen concentrate vs. cryoprecipitate remain unclear. This meta-analysis compares the patient-important outcomes associated with the use of fibrinogen concentrate vs. cryoprecipitate for the management of acquired hypofibrinogenemia in cardiac surgery.

**Methods:**

Medline, Embase, Cochrane Library, and Transfusion Evidence Library were searched from their inception until June 2024. Eligible studies included randomized clinical trials (RCTs). Effect estimates were synthesized using risk ratios (RR) and standardized mean differences (SMD), along with their corresponding 95% confidence intervals (CIs).

**Results:**

We analyzed 4 RCTs (945 participants: 823 adults, 122 children) comparing fibrinogen concentrate with cryoprecipitate undergoing cardiac surgery. Meta-analysis showed no difference in mortality (RR = 1.25, 95% CI: 0.79–1.96; moderate GRADE), blood loss (SMD = −0.14, 95% CI: −0.46–0.18), transfusion rates (blood cells: RR = 0.98, 0.77–1.26; platelets: RR = 0.17, 0.02–1.40; fresh frozen plasma: RR = 0.48, 0.16–1.45; cryoprecipitate: RR = 1.02, 0.58–1.81), infections (RR = 0.91, 0.64–1.28), volume overload (RR = 1.95, 0.18–21.34), transfusion reactions (RR = 0.98, 0.06–15.54), or postoperative thrombosis (RR = 0.76, 0.47–1.22). No allergic reactions were reported. Subgroup analysis revealed substantial heterogeneity (I^2^ = 0% to 98%) in most outcome measures between adults and children. Using the GRADE criteria, we assessed the quality of the evidence for mortality as moderate, whereas the quality of evidence for other outcomes was judged to be low.

**Conclusions:**

For patients undergoing cardiac surgery who experience clinically significant bleeding and hypofibrinogenemia, the available trial data provide moderate evidence that fibrinogen concentrate, compared to cryoprecipitate, does not increase the short-term risk of all-cause mortality. However, for the rate of transfusion of allogeneic or individual blood components, and adverse events, the existing evidence is of low certainty. Given the relatively small sample size, the group of children may not be representative of all children.

**Systematic Review Registration:**

(https://www.who.int/clinical-trials-registry-platform), identifier CRD42023421670.

## 1 Introduction

Excessive bleeding during cardiopulmonary bypass (CPB) remains a significant challenge in cardiac surgery and contributes to increased morbidity, mortality, and healthcare costs. Approximately 10% of patients undergoing cardiac surgery experience serious or massive bleeding, defined as chest tube output exceeding 1,000 ml within the first 12 h postoperatively (1–4). Despite significant differences in the hemostatic mechanisms between adults and infants (5–7), preoperative and postoperative plasma fibrinogen levels were negatively correlated with the occurrence of postoperative bleeding in both populations (8–10). One of the key determinants of successful hemostasis during and after CPB is the restoration and maintenance of normal fibrinogen levels, a crucial coagulation factor that is often depleted due to hemodilution, consumption, and degradation. Targeted supplementation may be considered in actively bleeding patients with laboratory-confirmed hypofibrinogenemia (11–13).

Theoretically, three methods can be used to elevate fibrinogen levels: Fresh Frozen Plasma (FFP), cryoprecipitate, and fibrinogen concentrate. However, mathematical models indicate that using FFP to increase fibrinogen levels above 1.5 g/L is impractical, leaving only cryoprecipitate and fibrinogen concentrate as viable options (14). Cryoprecipitate is composed of insoluble coagulation factors (15). According to regulations by the U.S. Food and Drug Administration (FDA), cryoprecipitate contains Fibrinogen (at least 150 mg), Factor VIII (at least 80 IU), Factor XIII (at least 50–75 IU), von Willebrand factor (at least 100–150 IU) and fibronectin (15–17). Fibrinogen concentrate contains fibrinogen and lacks other coagulation proteins (15, 18–20).

Cryoprecipitate was initially developed by Pool et al. in 1964 as a source of concentrated antihemophilic factor (Factor VIII) for treating hemophilia A (21) and has been in use for 60 years to date. Typically, it is prepared as a small pooled product derived from multiple donors rather than being distributed in single units (22). Cryoprecipitate can be stored frozen for up to one year. After thawing, its shelf life has conventionally been limited to 4–6 h due to concerns about Factor VIII degradation. However, fibrinogen remains stable for extended periods: *in vitro* studies confirm fibrinogen activity preservation for ≥5 days at 1–6 °C, and clinical protocols in some regions permit refrigerated storage for 72–120 h post-thaw (23, 24). Fibrinogen concentrate provides an alternative to cryoprecipitate for fibrinogen supplementation therapy and is amenable to commercial production (22). These products are lyophilized, highly purified, pathogen-reduced, and have a standardized fibrinogen content, allowing for rapid and precise dosing (22). Fibrinogen concentrate has a shelf life of up to three years at room temperature before reconstitution. Owing to variations in licensing, guideline recommendations, and clinical preferences, the use of cryoprecipitate and fibrinogen concentrate exhibits regional characteristics worldwide. Consequently, the debate over whether fibrinogen concentrate can replace cryoprecipitate remains unresolved (25–27).

The most recent systematic review that directly compared fibrinogen concentrate and cryoprecipitate was published in 2016(28). That review included only one study and did not thoroughly explore the efficacy and safety of these treatments nor has there been a plan for subsequent updates (28). Considering the recent advancements in this research field and the hazards associated with unsafe blood products (29–31), updating this systematic review is essential.

Therefore, we conducted a systematic review and meta-analysis to assess the evidence suggesting that fibrinogen concentrate is beneficial or harmful for patients with bleeding compared with cryoprecipitate. Additionally, we used GRADE analysis to determine the level of certainty of evidence for each outcome.

## 2 Methods

This systematic review and meta-analysis was conducted according to the Preferred Reporting Items for Systematic Review and Meta-Analyses (PRISMA) guidelines (32). The protocol was registered in the International Prospective Register of Systematic Reviews (PROSPERO; registration no. CRD42023421670) on May 22, 2023.

## 3 Search strategy

We systematically searched the literature using the following electronic databases: MEDLINE via Ovid (1946–June 2024), Embase via Ovid (1974–June 2024), Cochrane Library (CENTRAL; 2024, Issue 7), and Transfusion Evidence Library (1950–June 2024). No language restrictions were applied. Additionally, we searched the national and international trial registries for unpublished or ongoing trials (ClinicalTrials.gov, https://www.who.int/clinical-trials-registry-platform). To find additional studies, we manually searched the reference lists of the articles selected for inclusion in this review. For our detailed search strategy, please refer to Supplementary Material 1.

## 4 Eligibility criteria

Randomized controlled trials (RCTs) were included if they reported at least one of the outcomes in patients who underwent CBP.

Studies that did not provide estimates or sufficient data to calculate estimates were excluded. Conference proceedings, abstracts, reviews, and commentaries were also excluded. Studies published in any language were included, but were excluded if we could not translate the articles. For studies with multiple manuscripts, the manuscript with the most comprehensive dataset was included, while the others were excluded (duplicate data).

## 5 Study selection

Endnote 20 was used to manage the study selection. The “Find Duplicates” command was used to identify and eliminate duplicate records. Two reviewers (ZCY and CY) independently assessed the records identified in the search to determine their eligibility. Disagreements between the reviewers were resolved through discussion or consultation with a third reviewer (YWJ). During title and abstract screening, references included by at least one reviewer were included for full-text screening to decrease the risk of incorrectly excluding studies.

### 5.1 Data collection process

Two blinded reviewers (CY and YWJ) used a standardized form (Microsoft Excel) for data extraction from the included studies. Any discrepancies between reviewers were resolved by a third reviewer (KDY).

### 5.2 Variables

The extracted information for each study included reviewer source (study ID, review author ID, date of extraction, contact author's details), study identification (author, setting, country), characteristics of participants (inclusion and exclusion criteria, total number of study participants, mean age of participants, and percentage of male participants), and primary and secondary outcomes.

### 5.3 Outcomes

Systematic reviews should include outcomes that are likely to be meaningful to the intended users and recipients of the reviewed evidence (33). Owing to limitations in personnel, funding, and ethics, this study focused only on core outcome sets that are meaningful to anticipated users. Core outcome sets for patients were identified from the clinician's perspective (as determined through consultations with WFJ, CY, and CTY, who have experience working in hematology).

The primary outcome was all-cause mortality. In instances where a published trial did not report mortality data, while the corresponding entry in the ClinicalTrials.gov registry did, the data from the registry were used. Secondary outcomes included blood loss, the rate of transfusion of allogeneic or individual blood components, and adverse events (AEs). AEs included infection, volume overload, transfusion reactions, thromboembolic events and allergic reactions.

To ensure a systematic and transparent approach to outcome classification, we aligned our outcome selection with the five core areas framework proposed by Ioannidis et al. for clinical trials (34). This framework categorizes outcomes into: (1) Death, (2) Physiological or clinical variables, (3) Life impact, (4) Resource use, and (5) Adverse events. In our analysis, all-cause mortality was classified under the “Death” domain and serves as a hard endpoint reflecting the ultimate clinical consequence of uncorrected hypofibrinogenemia; it is considered a measure of the ultimate effectiveness of correcting hypofibrinogenemia. Blood loss and transfusion-related measures were categorized under the “Physiological or Clinical Variables” domain, representing direct efficacy outcomes. Adverse events were reported under the “Adverse events” domain. This structured classification enhances the interpretability and methodological rigor of our findings.

If two or more studies reported on mechanical ventilation time, intensive care unit stay, and duration of hospitalization, we will conduct an exploratory analysis of these outcomes, which are categorized under the “Resource use” domain in the five-core-areas framework.

## 6 Risk of bias assessment

The quality of the study was independently evaluated by two reviewers (CY and CL) using the five domains defined by the Risk of Bias 2 (RoB 2) tool: the randomization process (35), deviations from intended interventions, missing outcome data, measurement of the outcome, and selection of the reported result. Any discrepancies between reviewers were resolved by a third reviewer (KDY).

## 7 Data analysis

Based on intention-to-treat (ITT) analysis, we performed our analyses using RevMan 5.4 software. For dichotomous variables, we applied Mantel-Haenszel random-effects models; for continuous variables, inverse variance random-effects models were used (36). For studies with zero events in one arm, RevMan automatically applies a 0.5 continuity correction to all cells of the 2 × 2 table when calculating risk ratios, as required for numerical stability (36). This adjustment is standard in most meta-analytic software for ratio-based effect measures (34). We analyzed the treatment effects in individual trials and reported the risk ratio (RR) for dichotomous data and standardized Mean Difference (SMD) for continuous data, with respective 95% confidence intervals (CI). Cochran's *χ*^2^ test and *I*^2^ statistic were used to examine statistical heterogeneity (36).

If studies reported median values and interquartile ranges, they were converted to mean and standard deviation to facilitate the calculation of SMD using methods proposed by Luo et al. and Wan et al. (37).

## 8 Subgroup analysis and investigation of heterogeneity

Considering the significant physiological differences in the hemostatic systems between adults and children (5–7), we planned to conduct subgroup analyses for adults and children to explore the heterogeneity of the results.

## 9 Sensitivity analysis

Given the potential for data on the cumulative amount of transfused allogeneic blood components (red blood cells, platelets, and plasma) or the amount of individual blood components to deviate from a normal distribution, we planned to perform a pooled analysis of these continuous indicators. This will help us to verify whether they are consistent with the trends observed in the binary outcome summaries.

## 10 Reporting bias assessment

To assess publication bias, if a meta-analysis included at least 10 trials, Egger's test and funnel plot were employed to examine publication bias (38).

## 11 Summary of findings and assessment of the certainty of the evidence

We used the GRADE approach to evaluate the quality of evidence and to calculate the absolute effects on several outcomes, including mortality, postoperative blood loss, postoperative transfusion rates of allogeneic blood components or individual blood components (red blood cells, platelets, and plasma), and adverse events. GRADEpro GDT was used to generate a summary of the findings in table (39).

## 12 Results

### 12.1 Results of the search

**Figure 1:**
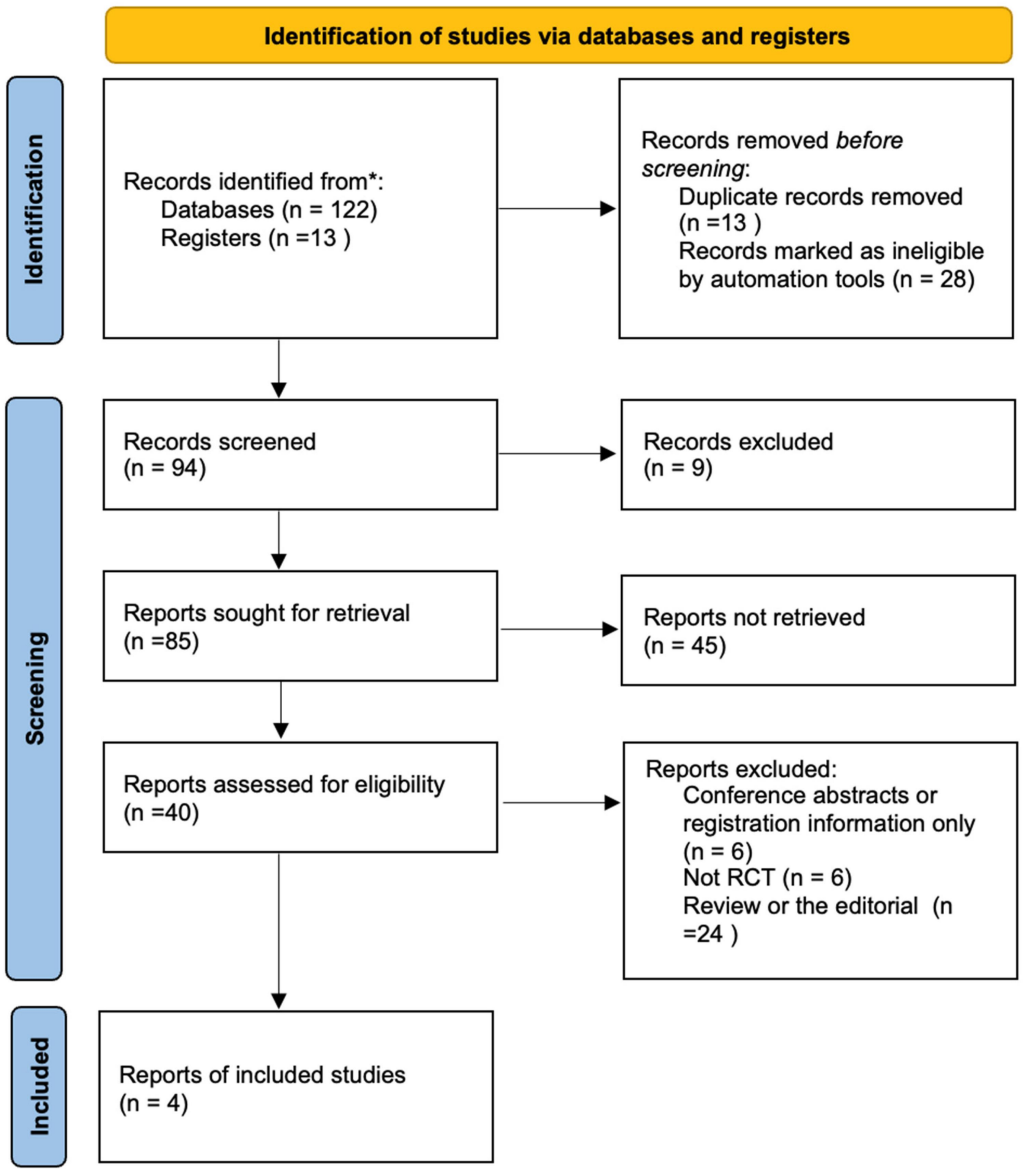
Flow chart of selection of studies on treatment effects of fibrinogen concentrates vs. cryoprecipitate with cardiopulmonary bypass.

A total of 135 records were identified in this study. After deduplication using the Endnote 20 software, 94 articles were retained. Compared to the Transfusion Evidence Library, the count was further reduced to 85. After reviewing titles and abstracts, 41 records were excluded [Two RCTs were excluded due to the use of a placebo as the control intervention (40, 41)]. We evaluated 44 articles obtained as full-text reports, and ultimately, 4 studies were included in the meta-analysis (Figure 1) (42–45).

### 12.2 Study characteristics

Four randomized controlled trials (RCTs) comparing fibrinogen concentrate treatment with cryoprecipitate (42–45). The range (years) of publication was 2014–2024. Two RCT encompassed 823 adult patients experiencing clinically significant bleeding and hypofibrinogenemia following cardiac surgery (42, 43). The remaining two RCTs involved 122 children undergoing non-emergent cardiac surgery with cardiopulmonary bypass (CPB) (44, 45). Two of the studies were published in developed countries (42, 45), whereas the other two were published in developing countries (43, 44). Three products were used as fibrinogen concentrates. Two studies employed Fibryga® (Octapharma) (43, 45), one utilized RiaSTAP® (CSL Behring) (44), and the other employed Haemocomplettan® P(CSL Behring) (45). The characteristics of the included studies are presented in the Table 1, and outcomes are shown in Table 2.

**Table 1:** The basic characteristics of included studies.

ID	Country	General information	Inclusion Criteria	Exclusion criteria	Interventions and controls	Funding
Callum et al. (43)	Canada	Adult; Median age = 64(years); %Male = 70.3; Fibrinogen Concentrate, *N* = 372; Cryoprecipitate, *N* = 363.	Fibrinogen plasma level <2.0 g/L by the Clauss method or FIBTEM [fibrin-based thromboelastometry test extrinsically activatedwith tissue fac tor and containing the platelet inhibitor cytochalasin D] derived clot amplitude at 10 min <10 mm by thromboelas tometry.	Receipt of fibrinogen concentrate or cryoprecipitate within 24 h before surgery; history of severe allergic reaction to fibrinogen concentrate or cryoprecipitate; refusal of blood components for religious or other reasons; plasma fibrinogen level greater than 3.0 g/L within 30 min of treatment order [to avoid increasing levels above the upper limit of normal (4.0 g/L)]; plasma fibrinogen level greater than 3.0 g/L within 30 min of treatment order [to avoid increasing levels above the upper limit of normal (4.0 g/L)];known pregnancy.	Fibrinogen Concentrate: Octapharma 4 g of fibrinogen concentrate (Fibryga; Octapharma AG); Cryoprecipitate: 10 units	Octapharma AG (Lachen,Switzerland)
Downey et al. (44)	US	≤1 years; Median age = 0.33(years); %Male = 59.3; Fibrinogen Concentrate, *N* = 30; Cryoprecipitate, *N* = 29.		gestational age (GA) of <32 weeks at birth and/or <36 weeks GA on the day of surgery (DOS); weight <3 kg on the DOS, emergency surgery; patient or family history of coagulopathy or thrombosis; active infection.	Fibrinogen Concentrate: RiaSTAP dose = (target fibrinogen level − measured fibrinogen level)/1.7 × weight (kg); Cryoprecipitate: 2 units	Stanford University (SPARK Foundation grant) and Children's Healthcare of Atlanta Foundation Grant.
Galas et al. (45)	Brazil	≤7 years; Median age = 0.29(years); %Male = 55.6; Fibrinogen Concentrate, *N* = 30; Cryoprecipitate, *N* = 33.		inability to receive blood products; enrolment in another study, chronic anaemia (preoperative haemoglobin <10 g dl^−1^); a past history of coagulopathy or preoperative coagulopathy (platelet count <100,000 ml mm^−3^ or prothrombin time >14.8 s); active infection or hypersensitivity to fibrinogen concentrate.	Fibrinogen Concentrate:Haemocomplettan® P 60 mg.kg^−1^; Cryoprecipitate: 10 ml.kg^−1^	No financial funding was received
Ayaganov et al. (42)	Kazakhstan	aged ≥ 18 years; Median age = 58.5 (years); %Male = 54.5; Fibrinogen Concentrate Group, *N* = 48; Cryoprecipitate Group, *N* = 40.	Significant hemorrhage; Hypofibrinogenemia, defined as fibrinogen plasma level; <200 mg/dl as confirmed by the Clauss method.	receipt of fibrinogen concentrate or cryoprecipitate within 24 h before surgery; history of severe allergic reaction to fibrinogen concentrate or cryoprecipitate; refusal of blood components for religious or other reasons.	Fibrinogen Concentrate:Octapharma Dose = (target fibrinogen level (mg/dl) − measured fibrinogen level (mg/dl)/1.8*weight (kg); Cryoprecipitate: 1 unit of cryoprecipitate per 5–10 kg body weight	Octapharma, Lachen, Switzerland

**Table 2:** Classification of outcomes according to the five-domain framework for clinical trials.

Core domain	Outcomes	Data type	Callum et al. (43)	Downey et al. (44)	Galas et al. (45)	Ayaganov (42)
Death	Mortality	Binary	√	√	√	√
Physiological or clinical variables	Cumulative transfusion volume of allogeneic blood products	Continuous (Units)	√	√	—	—
	Cumulative transfusion volume of Red Blood Cells	Continuous (Units)	√	√	—	—
	Cumulative transfusion volume of Platelets	Continuous (Units)	√	√	—	—
	Red Blood Cell transfusion rate	Binary	—	√	√	√
	Platelets transfusion rate	Binary	—	√	√	—
	Fresh frozen plasma transfusion rate	Binary	—	√	√	—
	Cryoprecipitate transfusion rate	Binary	—	√	√	—
	Blood losses	Continuous (ml)	—	—	√	√
Adverse events	Infection rate	Binary	√	√	—	√
	Volume Overload rate	Binary	√	—	—	√
	Transfusion reaction rate	Binary	√	—	—	√
	allergic reaction rate	Binary	√	—	—	√
	Postoperative thrombosis	Binary	√	√	√	√
Resource use	Duration of mechanical ventilation	Continuous (h)	√	√	√	—
	Intensive care unit stay	Continuous (days)	√	√	√	—
	Duration of hospitalization	Continuous (days)	√	√	√	—

### 12.3 Risk of bias in studies

Among the four mortality outcome trials, two studies adequately generated their randomization sequence, concealed allocation, and outcome assessment and were free from reporting bias (43, 45). Two studies did not describe the methods used for concealed allocation (42). One study did not describe the methods used to mask the participants (42). Two studies indicated that blinding clinicians involved in product administration was not feasible (43, 45), while the remaining two studies did not provide relevant information (42, 44). Additionally, two studies failed to specify whether outcome assessors were blinded (42, 44), and one study potentially exhibited selective reporting bias (42). Baseline characteristics were broadly consistent across the treatment groups in each trial. The risk of bias assessments and judgments are detailed in the Supplementary Material 2 in Table S2.

### 12.4 Outcomes

#### 12.4.1 Mortality

**Figure 2:**
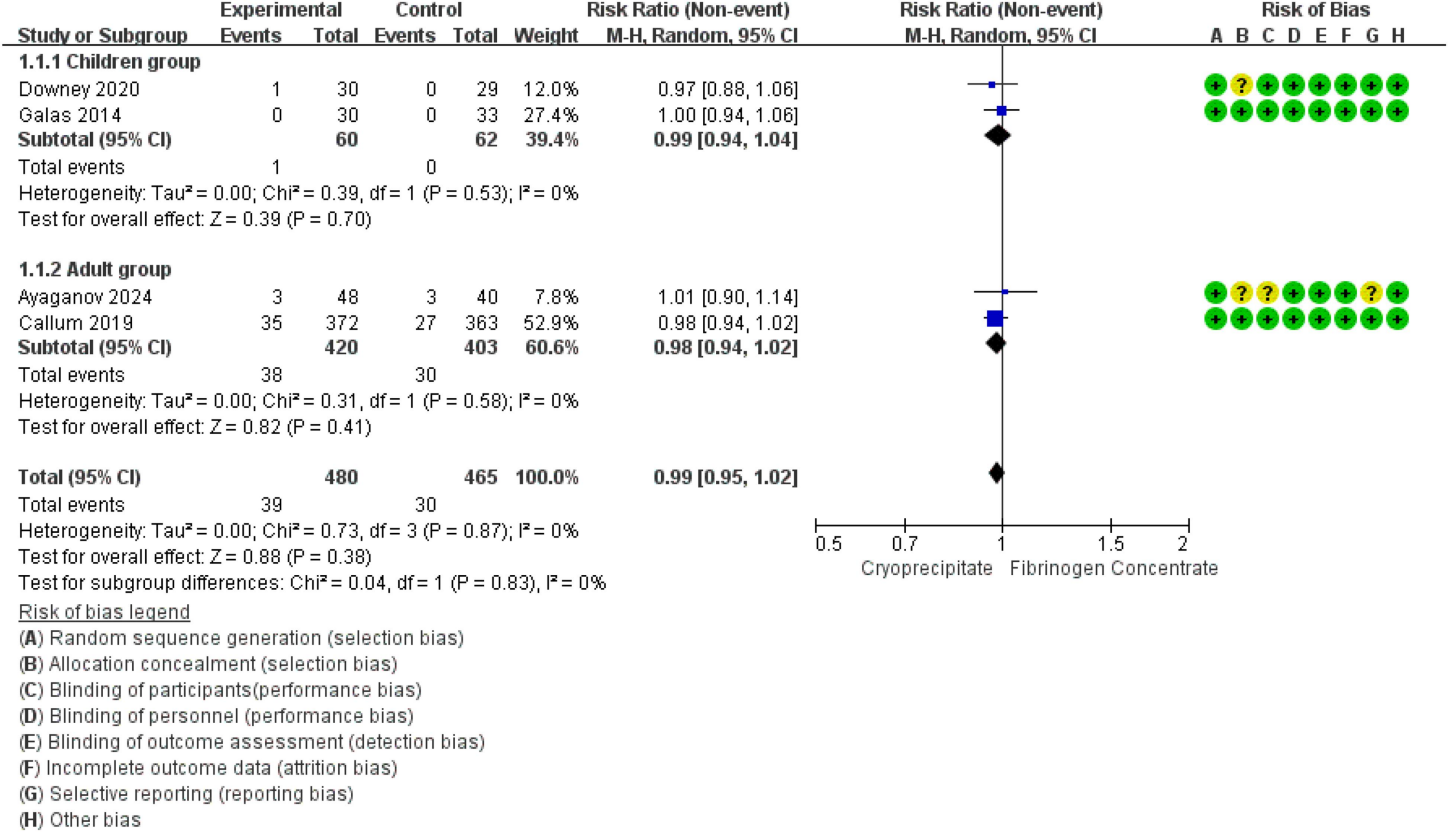
Forest plot: effects of fibrinogen concentrate vs. cryoprecipitate on all-cause mortality.

Meta-analysis of data from four RCTs (42–45) (945 participants, 122 children and 823 adults) suggested that the use of fibrinogen concentrate or cryoprecipitate was not linked to differences in mortality rates, with a pooled RR of 1.25 (95% CI: 0.79–1.96). No heterogeneity was detected within or between studies (Figure 2).

##### 12.4.1.2 GRADE approach

For the outcome ‘mortality’, we categorized the lack of blinding of personnel and outcome assessment as a low risk of bias.

Using the GRADE approach, we assessed the certainty of the evidence to be ‘mortality’. We downgraded the evidence certainty by one level to determine the risk of bias. The details are presented in Supplementary Material 3.

#### 12.4.2 Blood losses

**Figure 3:**
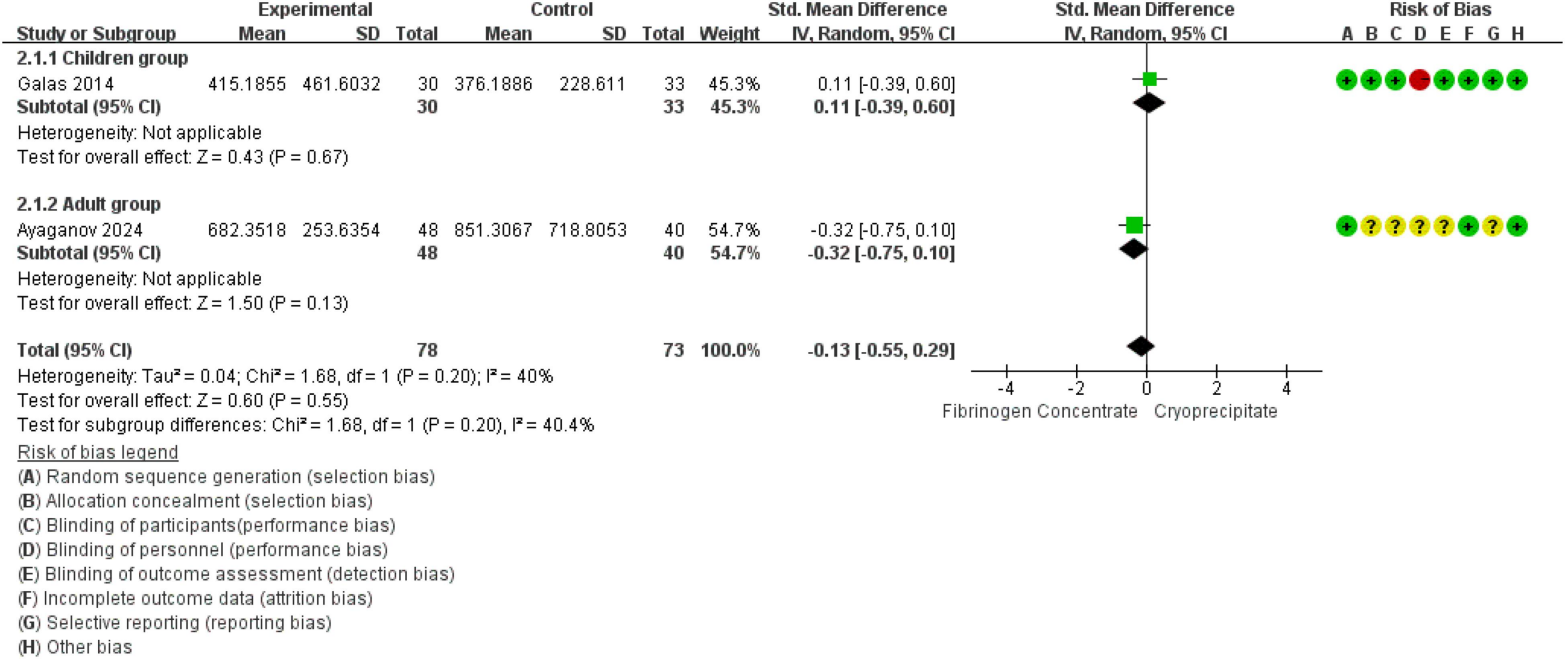
Forest plot: effects of fibrinogen concentrate vs. cryoprecipitate on blood losses.

Blood loss was reported in 2 RCTs (42, 45) (151 participants, 63 children and 88 adults). The pooled SMD was −0.14 (95% CI: −0.46 to 0.18), indicating that the differences were not statistically significant. Between-study heterogeneity was moderate (*I*^2^ = 40.4%) (Figure 3).

##### 12.4.2.1 GRADE approach

We assessed the certainty of the evidence to be ‘low’. We downgraded evidence certainty by three levels due to the following: risk of bias, indirectness and inconsistency. The details are presented in Supplementary Material 3.

#### 12.4.3 Allogeneic blood products transfused

**Figure 4:**
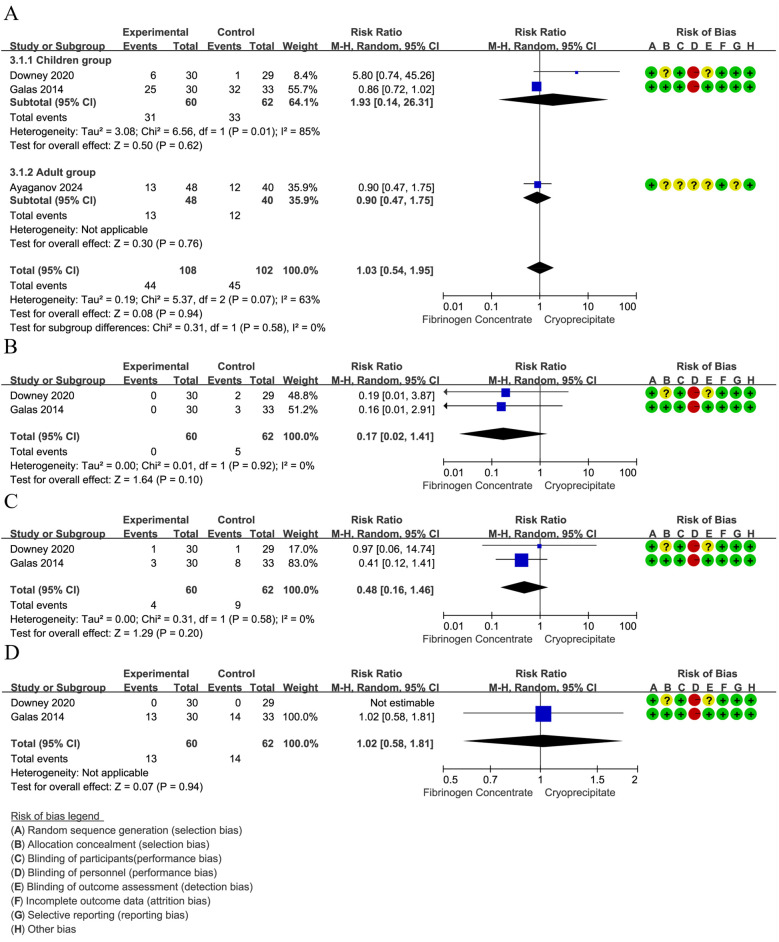
**(A)** Forest plot: effects of fibrinogen concentrate vs. cryoprecipitate on red blood cell transfusion rate. **(B)** Forest plot: effects of fibrinogen concentrate vs. cryoprecipitate on platelets transfusion rate. **(C)** Forest plot: effects of fibrinogen concentrate vs. cryoprecipitate on fresh frozen plasma transfusion rate. **(D)** Forest plot: effects of fibrinogen concentrate vs. cryoprecipitate on cryoprecipitate transfusion rate.

The red blood cell transfusion rate was reported in 3 RCTs (42, 44, 45) (210 participants, 102 children, and 88 adults). The pooled RR was 0.98 (95% CI: 0.77–1.26), indicating that the differences were not statistically significant. Notably, substantial heterogeneity emerged in the overall analysis (*I*^2^ = 63%), which was entirely resolved in subgroup stratification (*I*^2^ = 0%). Specifically, the high heterogeneity among studies involving children (*I*^2^ = 85%) suggests age-related differences in treatment response, whereas the adult studies showed low heterogeneity, indicating more consistent therapeutic effects in adults (Figure 4A). Pooled data from two RCTs (44, 45) in children (*n* = 122) revealed a non-significant trend toward reduced platelet transfusion requirements (RR = 0.17, 95% CI: 0.02–1.40). No heterogeneity was found between estimates (*I*^2^ = 0%) (Figure 4B). In children (two RCTs, *n* = 122) (44, 45), FFP utilization showed a non-significant 52% risk reduction (RR = 0.48, 95% CI: 0.16–1.45). No heterogeneity was found between estimates (*I*^2^ = 0%) (Figure 4C). In addition, analysis of two RCTs (44, 45) in children (*n* = 122) showed nearly identical cryoprecipitate transfusion rates between groups (RR = 1.02, 95% CI: 0.58–1.81).

##### 12.4.3.1 GRADE approach

We assessed the certainty of the evidence for the above four outcomes to be ‘low’. We downgraded evidence certainty by three levels due to the following: risk of bias, indirectness, inconsistency, and imprecision. The details are presented in Supplementary Material 3.

#### 12.4.4 Adverse events

**Figure 5:**
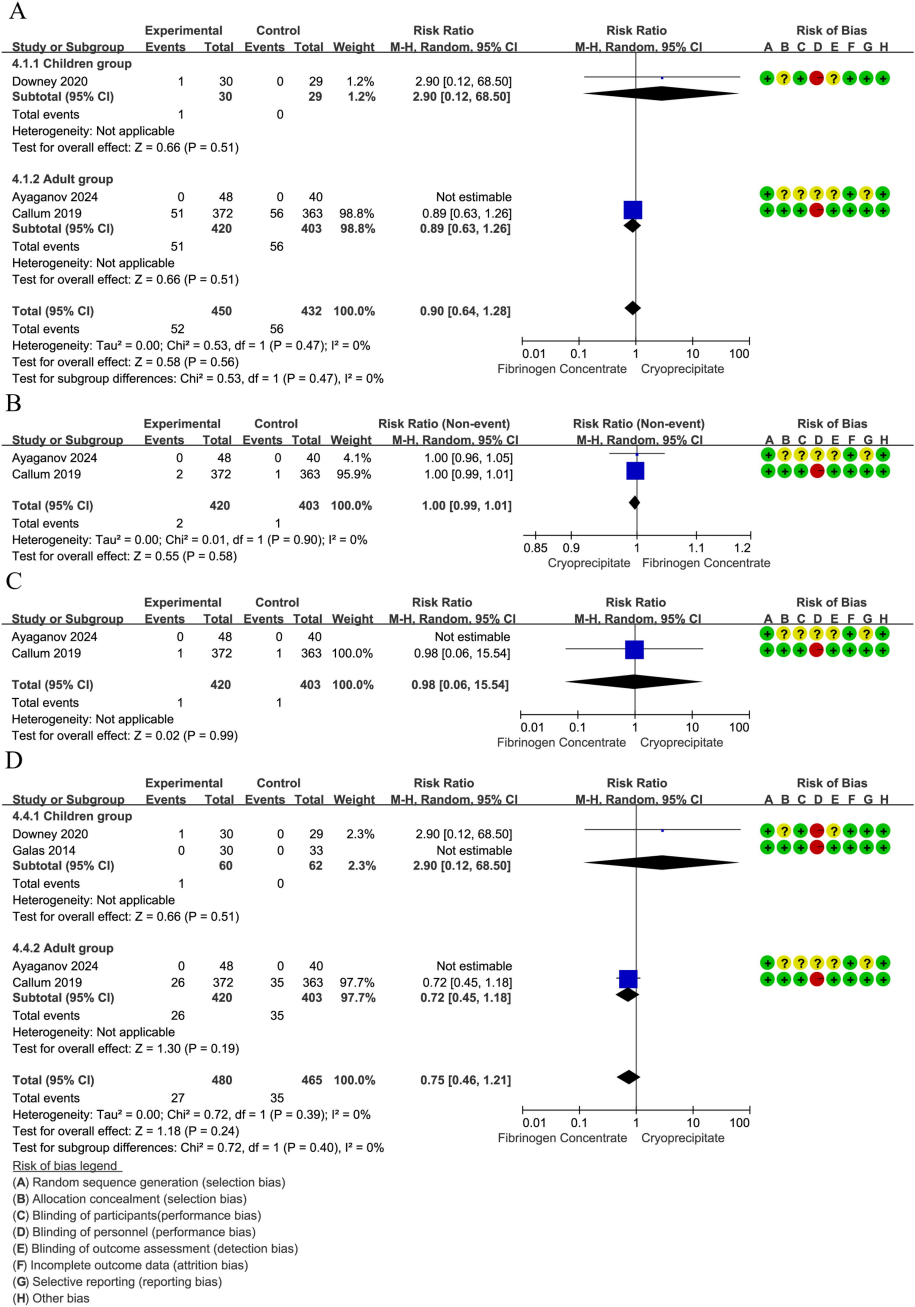
**(A)** Forest plot: effects of fibrinogen concentrate vs. cryoprecipitate on infection rate. **(B)** Forest plot: effects of fibrinogen concentrate vs. cryoprecipitate on volume overload. **(C)** Forest plot: effects of fibrinogen concentrate vs. cryoprecipitate on transfusion reactions. **(D)** Forest plot: effects of fibrinogen concentrate vs. cryoprecipitate on postoperative thrombosis.

The infection rate was reported in 3 RCTs (42–44) (882 participants, 59 children and 823 adults). The pooled RR was 0.91 (95% CI: 0.64–1.28), indicating that the differences were not statistically significant. No heterogeneity was found between the estimates (*I*^2^ = 0%) (Figure 5A). The Volume Overload rate was reported in two adults RCTs (*n* = 823) (42, 43). No statistically significant difference was observed between fibrinogen concentrate and cryoprecipitate (RR = 1.95, 95% CI: 0.18–21.34). Heterogeneity analysis was not applicable to this outcome (Figure 5B). The transfusion reaction rate was also reported in two adults RCTs (*n* = 823) (42, 43). The pooled RR was 0.98 (95% CI: 0.06–15.54), indicating that the differences were not statistically significant. Heterogeneity analysis was not applicable to this outcome (Figure 5C). Analysis spanning four RCTs (42–45) (945 participants, 122 children and 823 adults) showed a 25% non-significant thrombosis risk reduction (RR = 0.76, 95% CI: 0.47–1.22). No heterogeneity was found between the estimates (*I*^2^ = 0%) or subgroup analysis (*I*^2^ = 0%) (Figure 5D). The allergic reaction rate was reported in 2 RCTs (42, 45) (63 children and 88 adults), with no allergic reactions occurring.

##### 12.4.4.1 GRADE approach

We assess the certainty of evidence for the above four outcomes as “low” (Supplementary Material 3). We downgraded evidence certainty by three levels due to the following: risk of bias, indirectness, and imprecision.

### 12.5 Sensitivity analysis

A sensitivity analysis was conducted for three continuous outcome measures (2RCTs, 59 children and 735 adults) (43, 44): cumulative transfusion volume of platelets (SMD = −0.08, 95% CI: −0.22 to 0.07), red blood cells (SMD = −0.00, 95% CI: −0.14 to 0.14), and allogeneic blood products (SMD = −1.35, 95% CI: −3.96 to 1.25) (Supplementary Material 4).

### 12.6 Exploratory analysis

The exploratory analysis results (3RCTs, 122 children and 735 adults) (43–45) indicated that, compared to cryoprecipitate, fibrinogen concentrates did not increase mechanical ventilation time (SMD = −0.06, 95% CI: −0.35 to 0.22), intensive care unit stay (SMD = 0.04, 95% CI: −0.10 to 0.17), or Duration of hospitalization (SMD = −0.09, 95% CI: −0.22 to 0.04) (Supplementary Material 5).

## 13 Discussion

### 13.1 Summary of main results

Our study indicates that, compared to cryoprecipitate, fibrinogen concentrates do not increase the risk of post-surgery mortality, which is supported by moderate-quality evidence. It also did not increase the rates of red blood cell transfusion, platelet transfusion, fresh frozen plasma transfusion, cryoprecipitate transfusion, or postoperative thrombosis. Furthermore, fibrinogen does not increase the cumulative transfusion volume of allogeneic blood products and whole red blood cells nor does it reduce postoperative blood loss. However, the certainty of these findings remains low.

### 13.2 Overall completeness and applicability of evidence

We sought to identify and synthesize all existing RCTs to provide a comprehensive assessment of the differences in effectiveness and safety between fibrinogen concentrate and cryoprecipitate after cardiac surgery. These RCTs are relevant to the current clinical practice, as all included trials were conducted within the past decade across diverse settings (Kazakhstan, Brazil, Canada, and the United States). Cryoprecipitate has been in use for over 60 years, and although there are variations in the production techniques across these regions, the compositions are broadly similar (15–18, 22). Moreover, the fibrinogen concentrate products used in these studies were obtained from three commercial products from two companies, and are widely applied (Supplementary Material 6) (18, 19, 25). Our included studies included both adults and children, including preterm infants and babies. We conducted preplanned subgroup analyses and used the GRADE approach to determine the level of convincing evidence.

### 13.3 Limitation

Our study has some limitations. During the search for clinical trial registries, we identified a completed study that was excluded owing to the lack of critical information, potentially leading to biased estimates in our analysis. The study population included both adults (42, 43) and children (44, 45), and given that the children's hemostatic systems were not fully developed (5–7), we conducted subgroup analyses. The subgroup analysis for children included two studies (44, 45) with small sample sizes, leading to imprecision in our aggregated results. Although these studies performed sample-size estimations, their primary objectives differed from those of our study.

For the adult subgroup analysis, the sample size was significantly larger than that for the children subgroup, but there was also a substantial disparity in sample sizes between the two studies included, inevitably increasing the heterogeneity of the study population (42, 43). Clinical heterogeneity was also observed in the intervention and control measures. Our study involved three types of fibrinogen concentrate products, and research indicated that Fibryga® had a higher FXIII concentration than RiaSTAP® (46). Furthermore, there are variations in the dosage and administration methods (18–20). For fibrinogen concentrates, the administered dosages vary across different studies. Some studies calculate the dosage based on the patient's body weight (42, 44, 45), while others employ a fixed dose (43). For cryoprecipitates, regional differences in manufacturing processes also exist (15). Regarding outcome measures, owing to the specificity of this field, most continuous outcome measures were reported only as medians and ranges or medians and interquartile ranges, and we had to convert these data, which increased the imprecision of the results.

In addition, the primary outcome measure was all-cause mortality. Although the current research suggests a causal link between achieving safe and effective fibrinogen levels post-surgery and mortality, death following surgery is caused by complex circumstances and mechanisms. For instance, comorbidities and concomitant medications are associated with clinical outcomes and may increase surgical complexity, thereby prolonging operative duration. The length of surgery, in turn, can influence postoperative fibrinogen levels, concentrations of coagulation factors, the use of prophylactic antithrombotic agents, and the administration of cryoprecipitate postoperatively (25). Limited by the reported studies and preset meta-analysis protocols, we were unable to explore these aspects in depth. The outcomes related to physiological or clinical variables, adverse events, and resource use were assessed using heterogeneous definitions and measurement approaches across studies, which limits the interpretability of the results.

Lastly, the pooled estimates for adult outcomes were disproportionately weighted by a single large trial (Callum, 2019, *n* = 735) (43), which contributed 89% of the adult cohort. While sensitivity analyses showed consistent directionality, this imbalance may obscure potential effects in smaller patient subsets or distinct clinical scenarios.

## 14 Quality of the evidence

### 14.1 Methodology

Four studies reported methods for generating random sequences (42–45), but only two mentioned the key information about the implementation of allocation concealment (43, 45), which was judged as “low risk”. Regarding the blinding of participants, only one study lacked crucial information and were assessed as “unclear risk” (42), while the others were assessed as “low” (43–45). For the blinding of personnel, although challenging to achieve in this field, each study was evaluated based on the provided information; two was “High risk” (43, 45) and the others were “Unclear” (42, 44) Regarding the blinding of outcome assessment, one was “low risk” (42) and the others were deemed “Unclear” due to a lack of key information (43–45). The integrity of the results and selective reporting were considered low or unclear risk. Overall, most of the bias risks were categorized as low or unclear.

### 14.2 Outcome assessment

#### 14.2.1 Primary outcome

Four studies reported objective outcomes of mortality. Two of these studies accurately reported their follow-up durations (43, 44), whereas the follow-up durations in the other two studies could not be precisely determined (42, 45), and the follow-up periods were classified as post-CPB surgery.

#### 14.2.2 Secondary outcomes and exploratory outcomes

For the outcome of blood loss, two studies were included, that exhibited heterogeneity in the definition and follow-up duration were included (42, 45). Regarding the rate of transfusion of allogeneic blood components or individual blood components, the number of studies varied for each parameter, and there was heterogeneity in definitions and follow-up durations for these parameters (42–45). For the outcome of mechanical ventilation, three studies were included (43–45), and the original data were transformed to account for the variability in ventilation modes and differing respiratory needs of patients at various times. The lack of key information has led to inconsistencies in the definition of this outcome. In terms of adverse events, the number of studies and definitions varied, showing heterogeneity in definitions and follow-up times (42–45). The study by Callum et al. provided a detailed report on the definition of adverse events (43), whereas Ayaganov et al. included the least detail (42). Regarding ICU and hospital Length of Stay metrics, Three studies were included (43–45). It was noted that these metrics might vary owing to different standards across hospitals, leading to heterogeneity in definitions. These findings highlight the challenges in achieving consistency across studies owing to varying definitions and follow-up times in clinical research.

The outcome assessors were not blinded. Although most outcomes assessed are objective by nature, it is conceivable that all outcomes, except mortality, could be influenced by the lack of blinding. This lack of blinding introduces a potential bias that might affect the validity of the study results for these particular outcomes.

### 14.3 Heterogeneity

In addition to the clinical heterogeneity mentioned in the limitations section, we did not observe statistical heterogeneity in most outcomes, which may be due to the limited number of studies and inadequate reporting. Considering the differences between the hemostatic systems in adults and children, we conducted subgroup analyses in which heterogeneity was not observed.

#### 14.3.1 GRADE approach

We used the GRADE methodology to assess the certainty of evidence for various outcomes: mortality, blood loss, red blood cell transfusion rate, platelet transfusion rate, fresh frozen plasma transfusion rate, cryoprecipitate transfusion rate, infections, volume overload, transfusion reactions and postoperative thrombosis (Supplementary Material 3 details in Table 3). According to the GRADE criteria (39), the evidence quality for mortality was moderate, whereas it was low for all the other assessed outcomes.

**Table 3:** The GRADE system classifies the quality of evidence in one of four grades.

Grade	Definition	Outcomes
High	Further research is very unlikely to change our confidence in the estimate of effect	
Moderate	Further research is likely to have an impact on our confidence in the estimate of effect and may change the estimate	Mortality
Low	Further research is very likely to have an important impact on our confidence on the estimate of effect and is likely to change the estimate	blood loss, red blood cell transfusion rate, platelet transfusion rate, fresh frozen plasma transfusion rate, cryoprecipitate transfusion rate, infections, volume overload, transfusion reactions and postoperative thrombosis
Very low	Any estimate of effect is very uncertain	

## 15 Potential biases in the review process

Although we are confident that our electronic and manual searches captured the most relevant trials, there remains the possibility that some applicable literature or unpublished studies may have been missed. Our manual search ceased in July 2024, and additional studies may have been published afterward. We plan to update this systematic review over two years. Due to the small number of studies, we were unable to assess publication bias using funnel plots and regression analysis.

Additionally, some outcomes are intermediary, such as infections that may prolong hospital stays and lead to death, or extended mechanical ventilation, which often indicates more severe illness and is also associated with mortality (47). However, these studies did not consider such competing risks, which may lead to a biased estimation of the mortality outcome. This highlights the need for cautious interpretation of the results and consideration of the underlying factors that might influence the outcomes beyond the direct measures reported.

## 16 Agreements and disagreements with other reviews

We noted another systematic review that was less stringent in its inclusion and exclusion criteria compared to ours, focused on different outcomes, and included only one study (28). Therefore, it is not straightforward to determine if the results of this systematic review are similar to ours.

Other reviews have discussed whether fibrinogen or cryoprecipitate compared to placebo or other treatments impact mortality and other outcomes (48). Similar to our review, these analyses also raise concerns about the quality of trials, heterogeneity of interventions, imprecision and publication bias. This finding suggests that the certainty of these findings is low and should be interpreted and applied with caution. These observations underscore the importance of rigorous methodologies and the need for careful assessment of evidence quality in systematic reviews.

## 17 Authors’ conclusions

### 17.1 Implications for practice

Current clinical evidence indicates that fibrinogen concentrate and cryoprecipitate demonstrate comparable efficacy and safety in the management of acquired hypofibrinogenemia following cardiopulmonary bypass.

Clinical decision-making should incorporate a comprehensive assessment of patient-specific factors, including comorbidities (e.g., renal impairment, thrombophilia), pregnancy status, nutritional status (albumin <3.0 g/dl), and religious objections to plasma-derived products. Contextual factors must also be considered, such as surgical urgency, institutional blood product availability, and real-time viscoelastic test results (particularly FIBTEM A5 <10 mm).

Product selection should further account for infusion volume limitations (e.g., maximum infusion rate ≤100 ml/h in patients with heart failure) and pathogen safety profiles, ensuring that the chosen therapy aligns with both patient needs and institutional capabilities.

### 17.2 Implications for research

Existing RCT data demonstrate that fibrinogen concentrate does not increase mortality risk compared with cryoprecipitate (moderate-certainty evidence). Similarly, no statistically significant differences were observed for other outcomes, though these were supported by low-certainty evidence. Given the limitations in evidence quality, we cannot exclude the possibility that future clinical studies may substantially impact these effect estimates. Ideally, this study should encompass all individuals with acquired hypofibrinogenemia post-CPB with clear definitions of exposure and outcomes, including details such as exposure time, dosage frequency, and timing of outcome measurements. Additionally, studies should focus on the correlation between substitute indicators post-CPB and mortality, the relationship between CPB surgery duration and outcomes, and incorporate survival analysis into the statistical plan. This approach offers a comprehensive understanding of the impact of treatment and its practical application in diverse clinical settings.

Additionally, a comprehensive economic evaluation should be conducted in the future. A study from the United States suggested that fibrinogen concentrate is more expensive than cryoprecipitate, even after adjusting for cryoprecipitate wastage (49). However, a “FIBERS” randomized controlled trial in Canada demonstrated that fibrinogen concentrate might be cost-effective for bleeding management in adult patients undergoing cardiac surgery who develop acquired hypofibrinogenemia due to bleeding (50). These two studies, conducted in different regions, had a gap of approximately seven years. Therefore, a forward-looking and comprehensive economic evaluation is essential.

At last, currently available fibrinogen concentrate products include Fibryga® (Octapharma AG, Lachen, Switzerland), RiaSTAP®/Haemocomplettan® P (CSL Behring GmbH, Marburg, Germany), FibClot®/Clottafact® (LFB, Les Ulis, France), Fibrinogen HT (Benesis, Osaka, Japan), FibroRAAS (Shanghai RAAS, Shanghai, China), and GCC-Fibrinogen (GC Pharma, Yongin, South Korea) (4). These products are not universally accessible, having received regulatory approval only in select regions (4). Different products may contain varying proportions of other components in addition to fibrinogen (4). Moreover, there is a lack of uniformity in clinical usage, including dosages and frequencies, across different regions. Given this heterogeneity in commercially available fibrinogen concentrates, real-world clinical studies addressing this topic are scientifically imperative.

## Data Availability

The original contributions presented in the study are included in the article/Supplementary Material, further inquiries can be directed to the corresponding authors.
