# Supplementary material for: Treatment effects of fibrinogen concentrates vs. cryoprecipitate for correcting hypofibrinogenemia in cardiac surgery patients: a systematic review and meta-analysis
Source: Front Cardiovasc Med. 2025 Oct 17;12:1671405. doi: 10.3389/fcvm.2025.1671405 (PMC12576893; doi:10.3389/fcvm.2025.1671405)
Supplement: Supplementary file 2 [file Datasheet1.docx]

**Supplemental Material**

**Supplement 1：search strategies**

**Medline**

1 exp Cardiopulmonary Bypass/

2 cardiopulmonary bypass.mp.

3 bypass, cardiopulmonary.mp.

4 bypass, heart-lung.mp.

5 bypasses, cardiopulmonary.mp.

6 bypasses, heart-lung.mp.

7 cardiopulmonary bypass.mp.

8 cardiopulmonary bypasses.mp.

9 heart lung bypass.mp.

10 heart-lung bypass.mp.

11 heart-lung bypasses.mp.

12 atriopulmonary shunt.mp.

13 cardiopulmonary shunt.mp.

14 1 or 2 or 3 or 4 or 5 or 6 or 7 or 8 or 9 or 10 or 11 or 12 or 13

15 cryoprecipitate.mp.

16 cryoprecipitated plasma.mp.

17 cryoprecipitated serum.mp.

18 fibrinogen concentrate.mp.

19 haemocomplettan.mp.

20 haemocomplettan p.mp.

21 riastap.mp.

22 15 or 16 or 17

23 18 or 19 or 20 or 21

24 22 and 23

25 14 and 24

**Embaes**

1 exp cardiopulmonary bypass/

2 cardiopulmonary bypass.mp.

3 bypass, cardiopulmonary.mp.

4 bypass, heart-lung.mp.

5 bypasses, cardiopulmonary.mp.

6 bypasses, heart-lung.mp.

7 cardiopulmonary bypass.mp.

8 cardiopulmonary bypasses.mp.

9 heart lung bypass.mp.

10 heart-lung bypass.mp.

11 heart-lung bypasses.mp.

12 atriopulmonary shunt.mp.

13 cardiopulmonary shunt.mp.

14 1 or 2 or 3 or 4 or 5 or 6 or 7 or 8 or 9 or 10 or 11 or 12 or 13

15 exp cryoprecipitate/

16 cryoprecipitated plasma.mp.

17 cryoprecipitate.mp.

18 cryoprecipitated serum.mp.

19 15 or 16 or 17 or 18

20 exp fibrinogen concentrate/

21 fibrinogen concentrate.mp.

22 haemocomplettan.mp.

23 haemocomplettan p.mp.

24 riastap.mp.

25 20 or 21 or 22 or 23 or 24

26 19 and 25

27 14 and 26

**Cochrane library**

1 exp Cardiopulmonary Bypass/

2 cardiopulmonary bypass.mp.

3 bypass, cardiopulmonary.mp.

4 bypass, heart-lung.mp.

5 bypasses, cardiopulmonary.mp.

6 bypasses, heart-lung.mp.

7 cardiopulmonary bypass.mp.

8 cardiopulmonary bypasses.mp.

9 heart lung bypass.mp.

10 heart-lung bypass.mp.

11 heart-lung bypasses.mp.

12 cardiopulmonary shunt.mp.

13 1 or 2 or 3 or 4 or 5 or 6 or 7 or 8 or 9 or 10 or 11 or 12

14 cryoprecipitate.mp.

15 cryoprecipitated plasma.mp.

16 cryoprecipitated serum.mp.

17 fibrinogen concentrate.mp.

18 haemocomplettan.mp.

19 haemocomplettan p.mp.

20 riastap.mp.

21 14 or 15 or 16

22 17 or 18 or 19 or 20

23 21 and 22

24 13 and 23

**Transfusion Evidence Library**

Cardiopulmonary bypass and cryoprecipitate and fibrinogen concentrate

**Supplement 2：Supplement 2: Surgical types and risk of bias**

Table 1. Distribution of Surgical Types in Adult and Children Patients

| **Patient Group** | **Study Source** | **Surgical Type** | **Fibrinogen concentrate** | **Cryoprecipitate** |
| --- | --- | --- | --- | --- |
| Adults | Callum 2019 | Aortic valve procedure | 165 (25.3%) | 146 (23.9%) |
|  |  | Aortic surgery | 161 (24.7%) | 177 (28.9%) |
|  |  | Coronary artery bypass grafting | 153 (23.4%) | 146 (23.9%) |
|  |  | Mitral valve procedure | 68 (10.4%) | 70 (11.4%) |
|  |  | Tricuspid valve procedure | 31 (4.7%) | 35 (5.7%) |
|  |  | Atrial/ventricular septal defect repair | 20 (3.1%) | 8 (1.3%) |
|  |  | Heart transplantation | 18 (2.8%) | 10 (1.6%) |
|  |  | Complex congenital heart surgery | 11 (1.7%) | 11 (1.8%) |
|  |  | Other | 26 (4.0%) | 9 (1.5%) |
|  | Ayaganov 2024 | Bentall-de Bono procedure | 5 (10.4%) | 6 (15.0%) |
|  |  | Coronary artery bypass grafting | 16 (33.3%) | 12 (30.0%) |
|  |  | HeartMate 3 left ventricular assist device implantation | 2 (4.2%) | 2 (5.0%) |
|  |  | Heart transplantation | 2 (4.2%) | 1 (2.5%) |
|  |  | Intimectomy | 1 (2.1%) | 0 (0.0%) |
|  |  | Intimothrombectomy from pulmonary arteries | 0 (0.0%) | 1 (2.5%) |
|  |  | Valsalva sinus plasty | 1 (2.1%) | 0 (0.0%) |
|  |  | Valve surgery | 20 (41.7%) | 18 (45.0%) |
|  |  | Valve surgery combined with coronary artery bypass grafting | 1 (2.1%) | 0 (0.0%) |
| Children | Galas 2014 | Correction of atrial or ventricular septal defects | 9 (30.0%) | 7 (21.2%) |
|  |  | Correction of single ventricle physiology (first, second, and third stage procedures) | 6 (20.0%) | 5 (15.2%) |
|  |  | Correction of left ventricular outflow tract obstruction | 1 (3.3%) | 6 (18.2%) |
|  |  | Correction of conotruncal cardiac anomalies | 9 (30.0%) | 12 (36.4%) |
|  |  | Other cardiac procedures | 5 (16.7%) | 3 (9.1%) |
|  | Downey 2020 | Aortic arch reconstruction | 0 (0.0%) | 1 (3.7%) |
|  |  | Arterial switch operation for transposition of the great arteries | 4 (13.8%) | 1 (3.7%) |
|  |  | Atrioventricular canal defect repair | 5 (17.2%) | 6 (22.2%) |
|  |  | Rastelli procedure for double-outlet right ventricle or tetralogy of Fallot with pulmonary stenosis | 1 (3.4%) | 3 (11.1%) |
|  |  | Septal defect repair | 6 (20.7%) | 4 (14.8%) |
|  |  | Repair of tetralogy of Fallot | 5 (17.2%) | 6 (22.2%) |
|  |  | Unifocalization of pulmonary arteries / Pulmonary artery plasty | 4 (13.8%) | 5 (18.5%) |
|  |  | Bidirectional superior cavopulmonary anastomosis (bidirectional Glenn shunt) | 3 (10.3%) | 1 (3.7%) |
|  |  | Blalock-Taussig shunt (systemic-to-pulmonary arterial shunt) | 1 (3.4%) | 0 (0.0%) |

Table 2. Risk of bias

| **Risk of bias** | **Callum 2019** | | **Downey 2020** | | **Galas 2014** | | **Ayaganov 2024** | |
| --- | --- | --- | --- | --- | --- | --- | --- | --- |
|  | **Authors' judgement** | **Support for judgement** | **Authors' judgement** | **Support for judgement** | **Authors' judgement** | **Support for judgement** | **Authors' judgement** | **Support for judgement** |
| **Random sequence genera tion (selection bias)** | low risk | Participants were randomly assigned (1:1) to study groups using a pseudorandom number generator (PROC PLAN procedure in SAS) in randomly permuted blocks of 4, stratified by center. | low risk | Computer-generated. | low risk | random-number table | low risk | Computer-generated |
| **Allocation concealment (selection bias)** | low risk | randomization schedule was kept at the blood banks in sequentially numbered opaque sealed envelopes (prepared by Ergomed GmbH), which were opened when the order for fibrinogen replacement was received. | unclear risk | Although it was mentioned that the anesthesiologist caring for the patient was aware of the group allocation, the parents, surgeons, and critical care physicians were not, specific details about the allocation were not clearly reported. | low risk | Opaque envelopes | unclear risk | Not described |
| **Blinding of participants(perfor mance bias)  All outcomes** | low risk | To maintain blinding, chart labels for both products stated “Fibrinogen Study Product4 grams.” | low risk | Not described. There was almost no impact on the measurement of objective outcomes. | low risk | Not described. There was almost no impact on the measurement of objective outcomes. | unclear risk | Not described.Based on the overall risk of bias assessment, the risk of bias was deemed unclear. |
| **Blinding of personnel (performance bias)  All outcomes** | High risk | Personnel are unlikely to be ade quately blinded with an intervention of this nature. | unclear risk | Not described. | High risk | The study was not able to blind all  personnel because it was not feasible to mask the assigned therapy. | unclear risk | Not described. |
| **Blinding of outcome assessment  (detection bias)** | low risk | Datacollectors and outcome assessors were blinded | unclear risk | Not described. | low risk | Outcome assessors was unaware of study-group assignments. | unclear risk | Not described. |
| **Incomplete outcome data  (attrition bias)** | low risk | Complete reporting | low risk | Complete reporting | low risk | Complete reporting | low risk | Complete reporting |
| **Selective reporting (reporting bias)** | low risk | Protocol available. All outcomes listed in methods were reported in the results. | low risk | No protocol available. All outcomes listed in methods were reported in the results. | low risk | No protocol available. All outcomes listed in methods were reported in the results. | unclear risk | No protocol available. Some outcomes listed in methods were insufficiently described. |
| **Other bias** | low risk | No evidence of baseline imbalance | low risk | No evidence of baseline imbalance | low risk | No evidence of baseline imbalance | low risk | No evidence of baseline imbalance |

**Supplement 3：GRADE**

| Outcomes | **Anticipated absolute effects^*^** (95% CI) | | Relative effect (95% CI) | № of participants (studies) | Certainty of the evidence (GRADE) |
| --- | --- | --- | --- | --- | --- |
|  | **Risk with cryoprecipitate** | **Risk with Fibrinogen concentrates** |  |  |  |
| Mortality | 65 per 1,000 | **64 per 1,000** (61 to 66) | **RR 1.25** (0.79 to 1.96) | 945 (4 RCTs) | ⨁⨁⨁◯ Moderate^a^ |
| Blood loss | - | SMD **0.14 lower** (0.46 lower to 0.18 higher) | - | 151 (2 RCTs) | ⨁◯◯◯ Very low^b^ |
| Red Blood Cell transfusion rate | 441 per 1,000 | **454 per 1,000** (238 to 860) | **RR 0.98** (0.77 to 1.26) | 210 (3 RCTs) | ⨁◯◯◯ Very low^c^ |
| Platelets transfusion rate | 81 per 1,000 | **14 per 1,000** (2 to 114) | **RR 0.17** (0.02 to 1.40) | 122 (2 RCTs) | ⨁◯◯◯ Very low^d^ |
| Fresh frozen plasma transfusion rate | 145 per 1,000 | **70 per 1,000** (23 to 212) | **RR 0.48** (0.16 to 1.45) | 122 (2 RCTs) | ⨁◯◯◯ Very low^d^ |
| Cryoprecipitate transfusion rate | 226 per 1,000 | **230 per 1,000** (131 to 409) | **RR 1.02** (0.58 to 1.81) | 122 (2 RCTs) | ⨁◯◯◯ Very low^d^ |
| Infections | 130 per 1,000 | **117 per 1,000** (83 to 166) | **RR 0.91** (0.64 to 1.28) | 882 (3 RCTs) | ⨁◯◯◯ Very low^d^ |
| Volume Overload | 2 per 1,000 | **5 per 1,000** (0 to 53) | **RR 1.95** (0.18 to 21.43) | 823 (2 RCTs) | ⨁◯◯◯ Very low^d^ |
| Transfusion reactions | 2 per 1,000 | **2 per 1,000** (0 to 39) | **RR 0.98** (0.06 to 15.54) | 823 (2 RCTs) | ⨁◯◯◯ Very low^d^ |
| Postoperative thrombosis | 75 per 1,000 | **57 per 1,000** (35 to 92) | **RR 0.76** (0.47 to 1.22) | 945 (4 RCTs) | ⨁◯◯◯ Very low^d^ |

Explanations:

a. Downgraded by one level due to the following: risk of bias (selection bias, performance bias, detection bias, and reporting bias).

b. Downgraded by three levels due to the following: risk of bias( selection bias, performance bias, detection bias, and reporting bias), indirectness( the definitions of outcome measures are ambiguous, and the follow-up durations for outcomes are not specified) and inconsistency (moderate heterogeneity).

c. Downgraded by three levels due to the following: risk of bias (selection bias, performance bias, detection bias, and reporting bias), indirectness (the definitions of outcome measures are ambiguous, and the follow-up durations for outcomes are not specified), inconsistency (substantial heterogeneity), imprecision (very few events occurred).

d. Downgraded by three levels due to the following: risk of bias( selection bias, performance bias, detection bias, and reporting bias), indirectness( the definitions of outcome measures are ambiguous, and the follow-up durations for outcomes are not specified), imprecision (very few events occurred).

**Supplement 4：Sensitivity Analysis**

**Cumulative transfusion volume of platelets**

Two studies reported the cumulative transfusion volume of platelets (794 participants, 59 children, 735 adults). The pooled SMD is -0.08 (95% CI, -0.22 to 0.07), indicating that the differences were not statistically significant. Heterogeneity between estimates was not applicable (Figure 1).


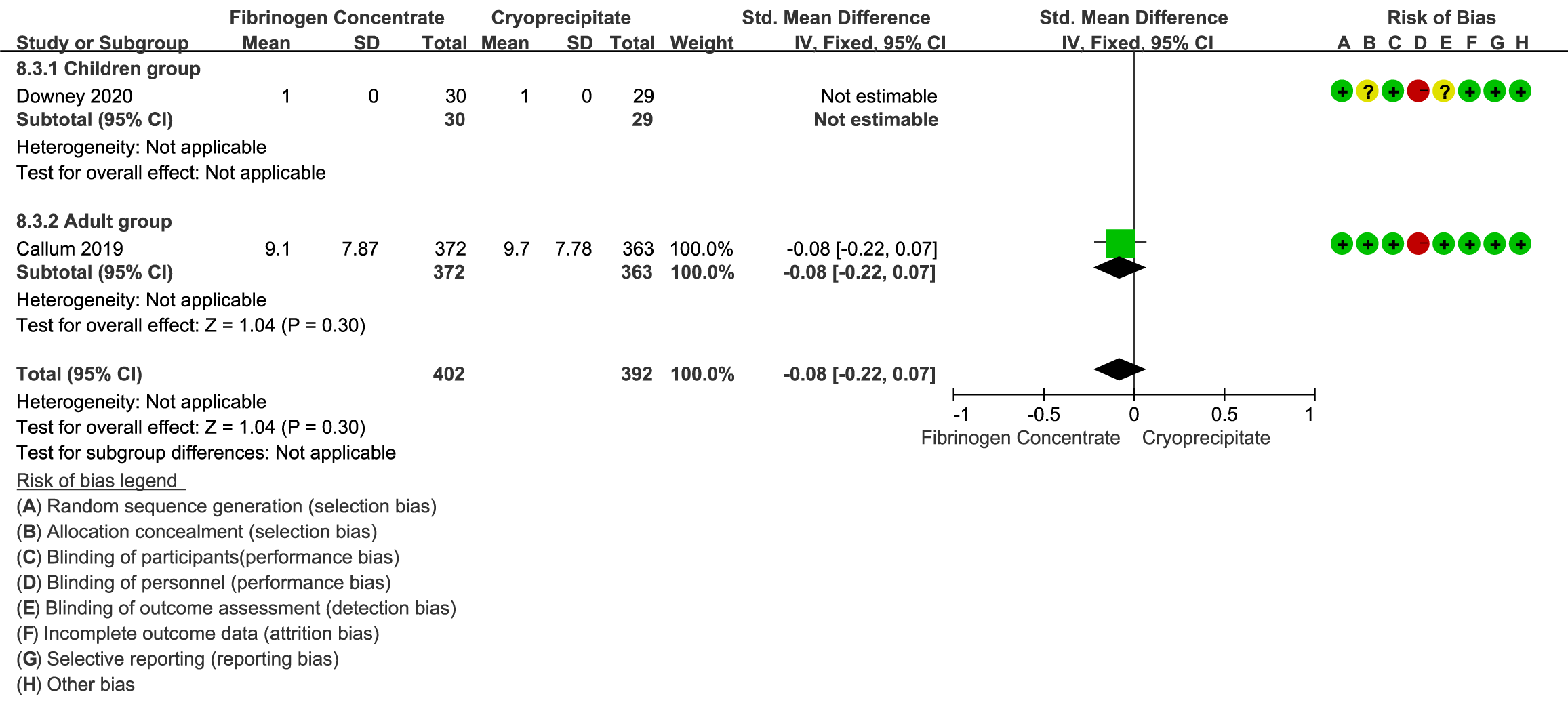


Figure 1. Forest plot: effects of fibrinogen concentrate versus cryoprecipitate on cumulative transfusion volume of platelet.

**Cumulative transfusion volume of** **red blood cell**

Two studies reported the cumulative transfusion volume of red blood cells (794 participants, 59 children and 735 adult). The pooled SMD is 0.00 (95% CI, -0.14 to 0.14), indicating that the differences were not statistically significant. Heterogeneity between estimates was not applicable (Figure 2).


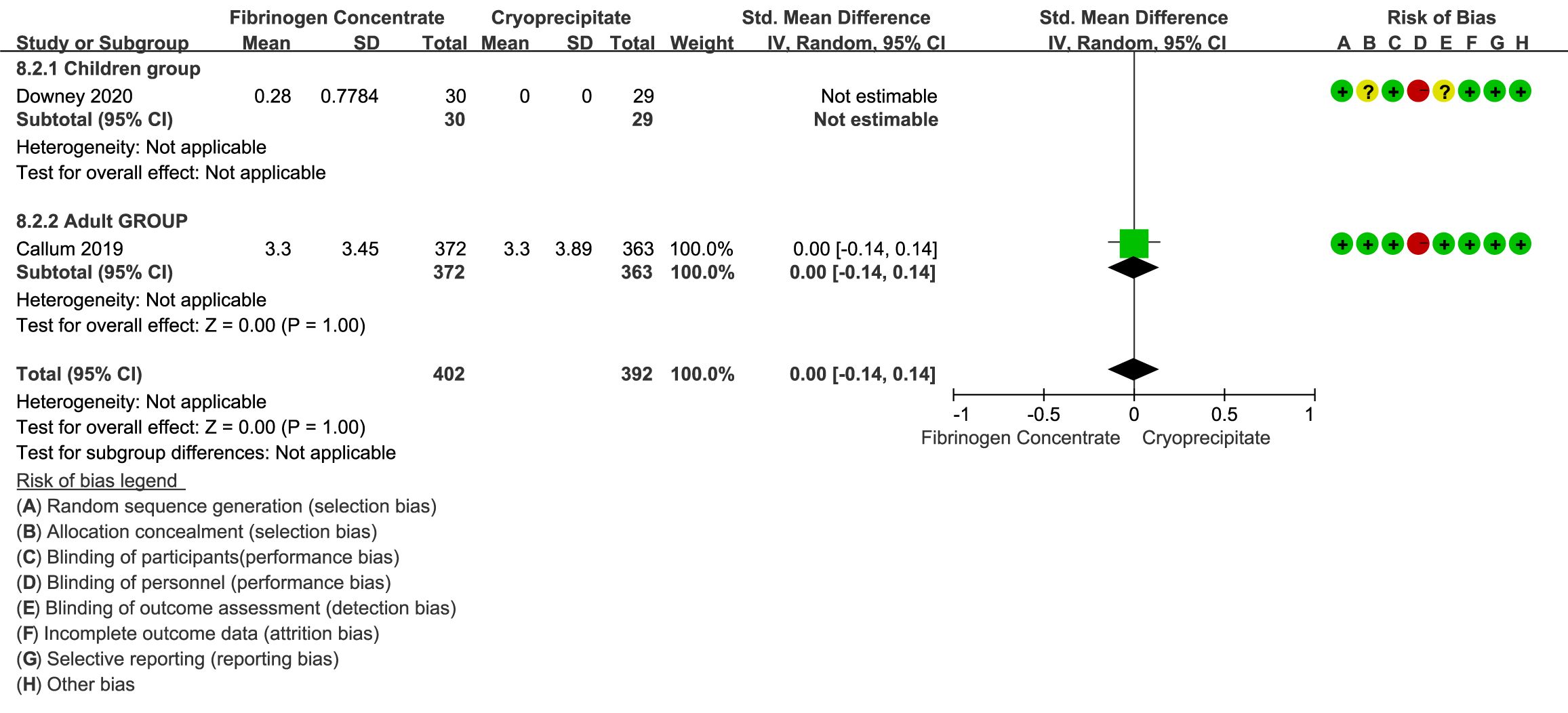


Figure 2. Forest plot: effects of fibrinogen concentrate versus cryoprecipitate on cumulative transfusion volume of red blood cell.

**Cumulative transfusion volume of allogeneic blood products**

Two studies reported the cumulative transfusion volume of allogeneic blood products (794 participants, 59 children and 735 adult). The pooled SMD is -1.35 (95% CI, -3.94 to 1.25). Overall heterogeneity was considerable heterogeneity (I^2^ = 98%), of which all 98% of the variance was estimated to be due to between-study heterogeneity (Figure 3).


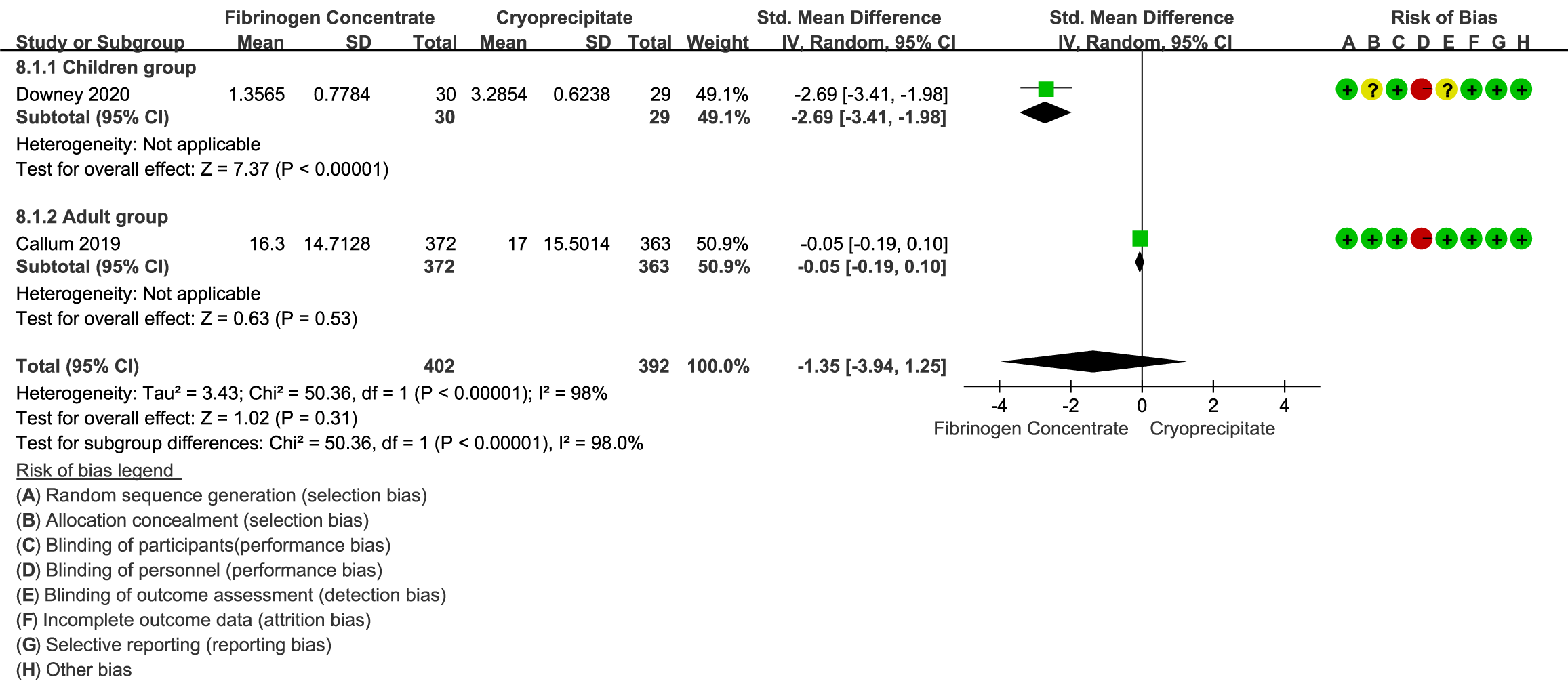


Figure 3. Forest plot: effects of fibrinogen concentrate versus cryoprecipitate on cumulative transfusion volume of allogeneic blood products.

**Supplement 5：Exploratory analysis**

**Duration of mechanical ventilation**

Duration of mechanical ventilation was reported in 3 RCTs (857 participants, 122 children, 735 adults). The pooled SMD was -0.06 (95%CI, -0.35 to 0.22), indicating that the differences were not statistically significant. Moderate heterogeneity was observed at the overall level (I²=46%). Subgroup analysis demonstrated substantial heterogeneity (I^2^=71.4%). In children group, no heterogeneity was observed (I^2^=0) (Figure 1).


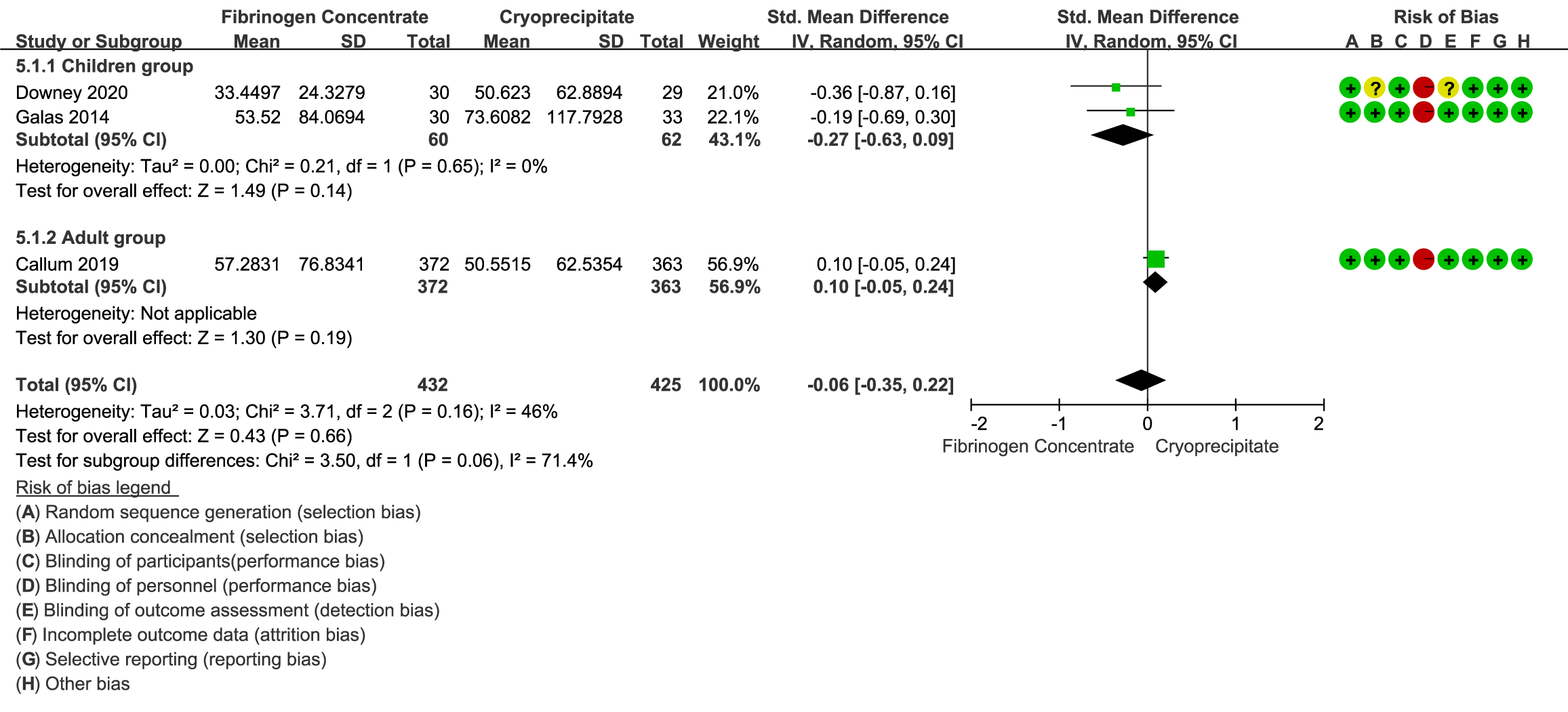


Figure 1. Forest plot: effects of fibrinogen concentrate versus cryoprecipitate on duration of mechanical ventilation.

**Intensive care unit stay**

Intensive care unit stay was reported in 3 RCTs (857 participants, 122 children and 735 adults). The pooled SMD was 0.04 (95% CI, -0.10 to 0.17), indicating that the differences were not statistically significant. No heterogeneity was found between estimates (I²= 0%) (Figure 2) .


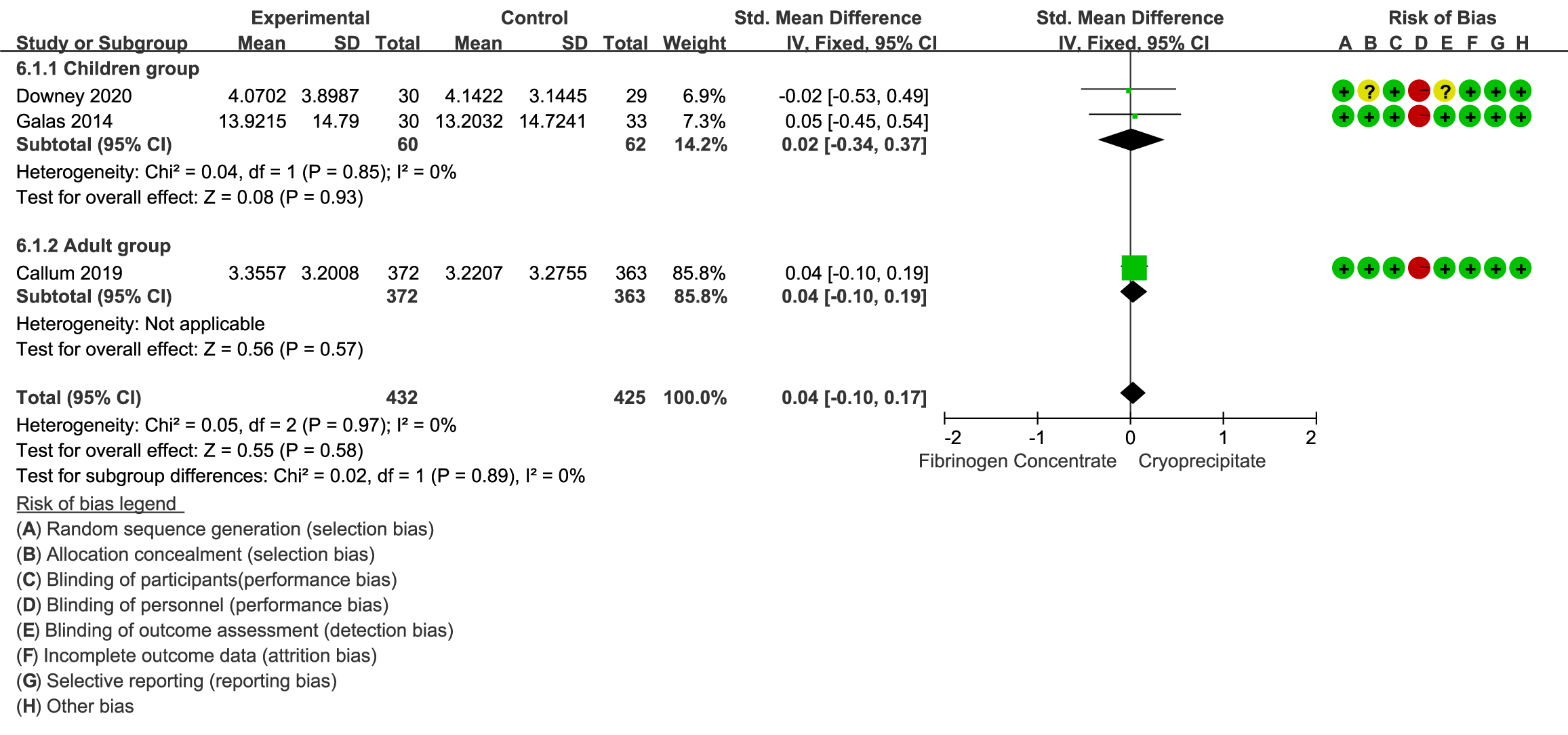


Figure 2. Forest plot: effects of fibrinogen concentrate versus cryoprecipitate on intensive care unit stay.

**Duration of hospitalization**

Duration of hospitalization was reported in 3 RCTs (857 participants, 122 children and 735 adults). The pooled SMD was -0.09 (95% CI, -0.22 to 0.04), indicating that the differences were not statistically significant. No heterogeneity was found between estimates (I² = 0%) (Figure 3) .


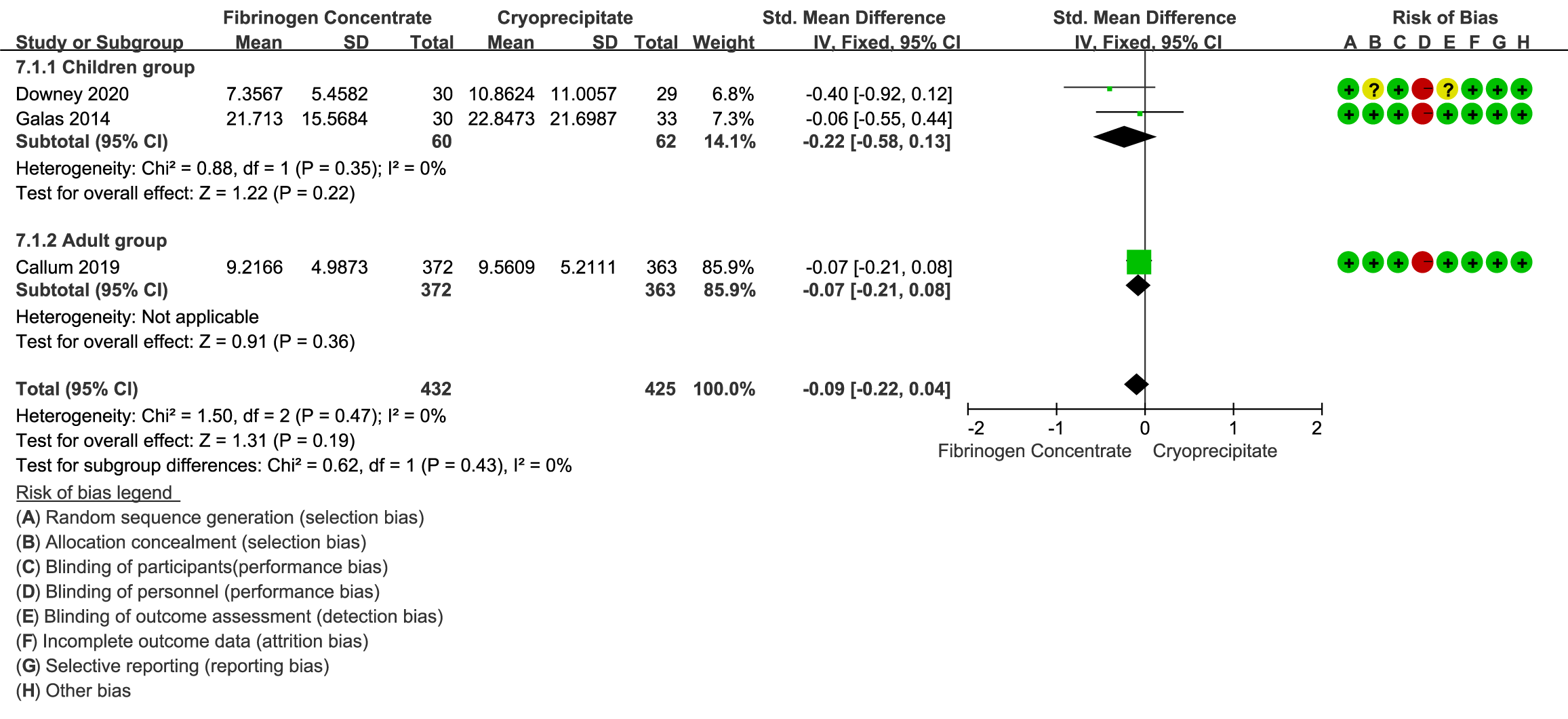


Figure 3. Forest plot: effects of fibrinogen concentrate versus cryoprecipitate on duration of hospitalization.

**Supplement 6：Pharmacokinetic (PK) Parameters for 3 FC products**

| Variable | Fibryga® | RiaSTAP® | Haemocomplettan® |
| --- | --- | --- | --- |
|  | Mean ± SD (range) | Mean ± SD  /Median (range) | Median (range) |
| Terminal t_1/2_ (h) | 75.9± 23.8 (40.0–157.0) | 78.7 ± 18.13/  77.1 (55.73-117.26) | 77.1 (55.73 – 117.26) |
| C_max_ (g/L) | 1.39 ± 0.369 (0.83–2.16) | 1.4 ± 0.27 /1.3 (1.00-2.10) | 1.3 (1.00 – 2.10) |
| AUC_norm_ | 1.62 ± 0.45 (0.85–2.51) |  |  |
| AUC^a^ (days·kg) h*mg/mL |  | 124.3 ± 24.16 /126.8 (81.73-156.40) | 126.8 (81.73 – 156.40) |
| CI (mL/h/kg) | 0.67 ± 0.20 (0.40–1.17) | 0.59 ± 0.13/0.55 (0.45-0.86) | 0.55 (0.45 – 0.86) |
| MRT (h) |  | 92.8 ± 20.11/85.9 (66.14-126.44) | 85.9 (66.14 – 126.44) |
| V_ss_ (mL/kg) | 70.2 ± 29.9 (36.9–149.1) | 52.7 ± 7.48/52.7 (36.22-67.67) | 1.7 (1.30 – 2.73) |
